# Impact of Light Conditions on Visual Performance following Premium Pseudophakic Presbyopia Corrections

**DOI:** 10.3390/jcm12134324

**Published:** 2023-06-27

**Authors:** Eirini-Kanella Panagiotopoulou, Kostas Boboridis, Ioannis Seimenis, Georgios Labiris

**Affiliations:** 1 Department of Ophthalmology, University Hospital of Alexandroupolis, 68100 Alexandroupolis, Greece; labiris@usa.net; 2 Ophthalmology Department, AHEPA University Hospital, 54636 Thessaloniki, Greece; kosbob@otenet.gr; 3 Medical Physics Laboratory, School of Medicine, National and Kapodistrian University of Athens, 11527 Athens, Greece; iseimen@med.uoa.gr

**Keywords:** activities of daily living, light intensity, light temperature, presbyopia, multifocal intraocular lens

## Abstract

The primary objective of this study was to objectively compare the visual performance of patients following premium pseudophakic presbyopia corrections in different light combinations for near- and intermediate-vision activities of daily living (ADLs). This is a prospective, comparative study. A total of 75 patients populated three study groups: G1-patients with bilateral trifocal implantation, G2-patients with bilateral bifocal implantation, and G3-patients with bilateral monofocal implantation. All participants addressed 10 ADLs in nine combinations of light temperature (3000 K, 4000 K, and 6000 K) and light intensity (25 fc, 50 fc, and 75 fc) and declared their subjectively optimal light combination while reading. G2 and G3 had the best total ADL scores in 6000 K/75 fc, while G1 had the best total ADL score in 4000 K/75 fc. Total ADL, easy ADL, and moderate difficulty ADL scores were significantly better in G2, while difficult ADL score was significantly better in G1. The majority of all groups selected 6000 K/75 fc as the most comfortable light combination, and no group selected 3000 K and 25 fc. In conclusion, trifocal patients benefit from intense daylight, while bifocal and monofocal patients benefit from intense, cold lighting. Trifocal patients present superior near-vision capacity in difficult near-vision daily tasks, while bifocal patients present superiority in easy and moderate-difficulty ADLs.

## 1. Introduction

Presbyopia is an age-related visual disorder that results in progressive near-vision impairment, reducing the ability mainly of emmetropic populations in their late 40s to perform near- and intermediate-vision activities of daily living (ADLs) [1,2]. Nowadays, working and social norms have been modified heavily by the usage of computers, tablets, and smartphones [3]. Moreover, since presbyopia primarily manifests in middle-aged people, it significantly reduces productivity and quality of life [4]. Several surgical interventions to address presbyopia have been introduced, such as laser-assisted corrections (i.e., Presby-LASIK, micro-anisometropia, etc.) [5,6,7,8,9], corneal inlays [10], and premium intraocular lens (IOL) implantations [11,12,13]. Among them, the most popular option is the removal of the crystalline lens and the implantation of a presbyopic IOL that attempts to restore the pre-presbyopic functionality of the eye. Presbyopic IOLs, including multifocal, extended-depth-of-focus (EDOF), and accommodative IOLs, can be implanted either in cataracts or in clear lens cases [14,15,16,17].

Two available multifocal IOLs are Panoptix (Alcon, FortWorth, TX, USA) and Restor +2.50 diopters (D) (Alcon, FortWorth, TX, USA). Panoptix is a trifocal, diffractive, nonapodized, yellow IOL of an acrylate/methacrylate copolymer. It has UV and blue radiation filters. Further to the distant focal point, it provides a near (at 40 cm/+3.20 D) and an intermediate focal point (at 60 cm/+2.20 D). Finally, it is supposed to provide optimal outcomes without being affected by pupil diameter [18]. Restor +2.50 D is a bifocal, hybrid (diffractive aspheric with a central refractive zone), apodized, yellow IOL of an acrylate/methacrylate copolymer. It has UV and blue radiation filters. In addition to distance vision, the added power of +2.5 D is designed to provide optimal vision at 53 cm for activities requiring intermediate vision [19].

According to their self-reported visual abilities, some people overestimate or underestimate their everyday functional abilities. Additionally, the estimation only of visual acuity cannot provide a comprehensive assessment of patients’ visual capacity. Therefore, ideally, any functional incapacity should be objectively and realistically evaluated for patients in their home or working environment by careful assessment of their performance in specific ADLs [20]. ADL performance varies based on the type of presbyopic corrections [21]. Specifically, it has been found that patients who underwent pseudophakic mini-monovision correction had lower performance in demanding ADLs, such as supermarket receipt reading, the search for an entry in a phonebook catalog, and reading of the print on a typical eye drop bottle in comparison with patients who underwent bilateral pseudophakic multifocal correction with implantation of bifocal hybrid IOLs [21].

Additionally, it has been shown that visual acuity is significantly correlated with ADL performance. Specifically, a validation study of a framework including ADLs that require near and intermediate vision capacity has proved that patients with perfect near visual acuity have the best ADL scores among three groups of near visual acuity, followed by the group with moderate and low near visual acuity [22]. Visual performance in different ADLs is also affected by lighting conditions. Specifically, a significant difference in ADL score has been revealed among three different lighting combinations of progressively higher light intensity and higher lighting color temperature (cooler light) in ADLs using printed material, while no difference has been revealed in almost all ADLs using screens or needing manual dexterity [22].

Moreover, it has been found that distance visual acuity increases with increasing light intensity. Additionally, the IOL type determines the minimum required illuminance level (MRIL). Specifically, patients implanted bilaterally with trifocal diffractive IOLs require significantly more illuminance than patients implanted bilaterally with monofocal IOLs to achieve the same visual acuity [23]. These differences in the MRIL arise from the different light transmission technology of each IOL type. For instance, Panoptix and Restor IOLs have an overall light transmittance ratio of 88% and 87.4%, respectively, which is almost identical to the light transmittance ratio of a monofocal IOL [24]. However, Panoptix’s trifocal design splits the light into three focal points, delivering 44% of the light to the distant focal point and 22% of the light to the intermediate and near ones [24]. On the other hand, Restor’s bifocal design splits the light into two focal points, delivering 69.4% of the light to the distance focal point and 18% of the light to the near one [25]. Therefore, the Panoptix and Restor light transmittance ratio for the near and/or intermediate focal points is lower than the one focal point of a monofocal IOL. As a result, it is expected that the visual performance of patients implanted with multifocal IOLs will also be affected by the lighting conditions.

Taking all the above into consideration, the question arises as to whether the visual performance in ADLs requiring near and intermediate visual capacity differs depending on the light temperature, light intensity, and IOL type. Within this context, the primary objective of this study was to compare the visual performance of patients following premium pseudophakic presbyopia corrections in an experimental lighting facility in different combinations of light intensity and light temperature for a series of ADLs that require near and intermediate vision.

## 2. Materials and Methods

### 2.1. Setting

This is a prospective, comparative study. The study protocol adhered to the Declaration of Helsinki, while written informed consent was provided by all participants. The scientific board of the Democritus University of Thrace approved the study protocol. The study was conducted at the Department of Ophthalmology in the University Hospital of Alexandroupolis, Greece, between September 2021 and September 2022. The official registration number of the study is NCT05359380.

### 2.2. Participants

Patients who visited our outpatient service were enrolled on a consecutive-if-eligible basis and populated the following three distinct study groups for the purposes of this study: (1) G1 group: patients that underwent bilateral presbyopic correction with the use of a trifocal diffractive IOL (PanOptix, Alcon Laboratories, Inc., Fort Worth, TX, USA), (2) G2 group: patients that underwent bilateral presbyopic correction with implantation of a bifocal hybrid IOL [ReSTOR (add +2.50) Alcon Laboratories, Inc., Fort Worth, TX, USA], and (3) G3 group: patients of similar age that underwent bilateral pseudophakic monofocal correction with implantation of an aspheric IOL (AcrySof IQ SF60WF Alcon Laboratories, Inc., Fort Worth, TX, USA).

Inclusion criteria for all groups included age older than 18 years old, diagnosis of senile cataract, either stage 2 or 3 of the Lens Opacities Classification System III (LOCS-3) grading scale, and fluency in the written and verbal Greek language, while exclusion criteria for all groups included: astigmatism >1.00 diopters, glaucoma, intraocular pressure-lowering medications, former incisional eye surgery, corneal or fundus disease, posterior capsule rupture or lens misalignment, autoimmune diseases, diabetes mellitus, neurological, psychiatric or mental diseases, and inability to understand the objectives of the study due to intellectual disability, low educational level, or low socioeconomic status.

For the minimization of potential dysphotopic phenomena in pseudophakic multifocal corrections, all G1 and G2 participants had to present pupil centroid shift (shift of the pupil center in the transition from the constricted pupil in photopic conditions to a dilated pupil in low mesopic conditions) lower than 0.4 mm and pupil diameter in low mesopic conditions smaller than 5 mm [26].

### 2.3. Surgical Technique

All surgical procedures were performed by the same surgeon (G.L.) in a consistent way, as described before, using the Centurion Vision System platform (Alcon, FortWorth, TX, USA) [15]. Pupil dilation was performed with tropicamide 0.5% (Tropixal, Demo, Krioneri, Greece), Phenylephrine Hydrochloride 5% (Phenylephrine, Cooper, Athens, Greece), and cyclopedolate (Cyclogyl, Alcon Laboratories, Athens, Greece). The periorbital skin and eyelids were cleaned, and the conjunctival sac was irrigated with a solution of povidone-iodine (Betadine). Patients received topical anesthesia with propacaine hydrochlorine 0.5% drops. All multifocal corrections were digitally guided with the Verion Image-Guided System (Alcon, FortWorth, TX, USA). Specifically, the main incision (2.2 mm self-sealing upper temporal or upper nasal), two contralateral stab incisions, capsulorhexis and IOL centration in the implantation of all multifocal IOLs, and IOL alignment in multifocal toric IOL implantation were guided by the Verion Digital Marker to ensure optimal outcomes. Digital guided capsulorhexis was set at 5.0 mm based on the visual axis. Then, 3% sodium hyaluronate and 4% chondroitin sulfate (Viscoat, Alcon, Athens, Greece) were injected for the phacofragmentation phase and 1% sodium hyaluronate (Provisc, Alcon, Athens, Greece) for the IOL implantation phase. In all G1 and G2 cases, the non-dominant eye was initially operated, followed by the dominant one within a time window of maximum 6 months. The same postoperative regimen was prescribed to all patients, which included (1) a fixed combination of tobramycin 0.3% and dexamethasone 0.1% (FCTD) (Tobradex, Alcon, Athens, Greece) six times daily, and (2) sodium hyaluronate 0.1% (Hylocomod, Pharmex, Athens, Greece) gradually tapered in a month.

### 2.4. Experimental Lighting Facility

**Figure 1:**
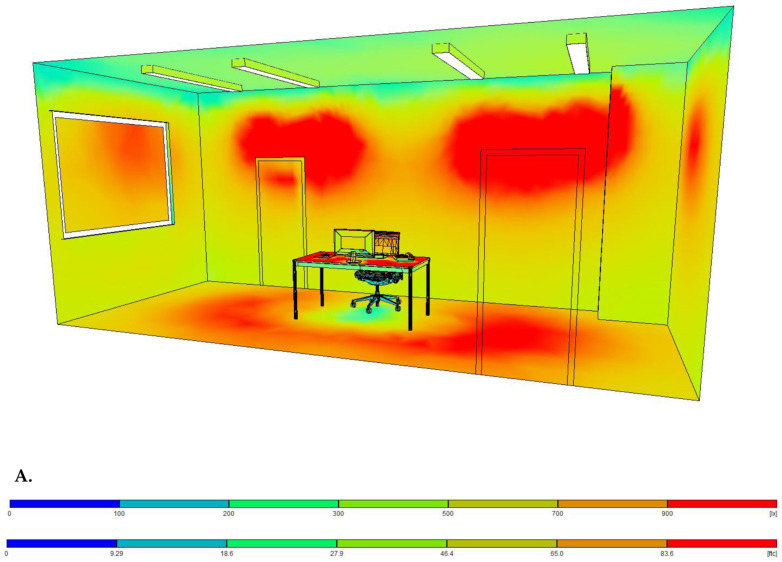

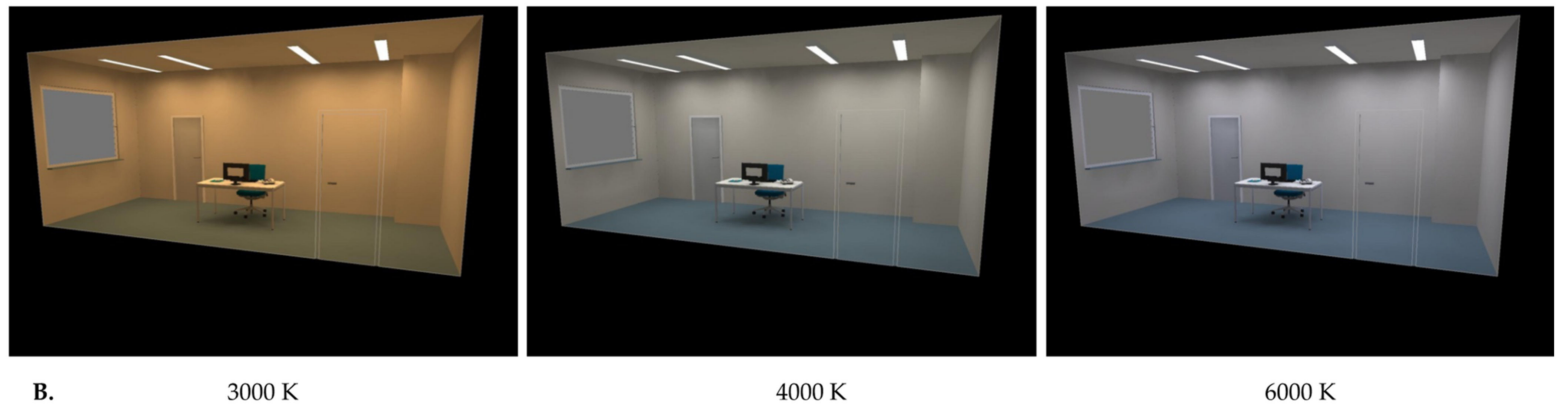
A three-dimensional (3D) illustration of the examination room with the four luminaires installed on the ceiling at their actual positions using the RELUX software. (**A**) The light intensity on the surfaces inside the room is overlaid and encoded in color according to the chromatic scale shown at the bottom (units: lx). (**B**) The three light temperatures (3000, 4000, and 6000 K) used are illustrated in a 3D simulation of the examination room.

The present study was conducted in a special experimental facility constructed at the University Hospital of Alexandroupolis, as described in the corresponding validation study [22]. An advanced light diffusion system consisting of four linear LED luminaires was installed in a hospital room with a dimension of 6.87 m × 2.9 m × 3 m (depth × width × height) and flat white surface walls (reflectance: 70%). Light intensity (dimming) and light temperature (white tuning) were adjusted using a wireless control application (Casambi Technologies Oy Inc., Espoo, Finland), which uses integrated Bluetooth mesh technology and secures maximal uniformity at different user-defined lighting settings. The four LED luminaires were mounted on the ceiling. The amount of the provided luminous flux and the exact luminaire positioning were defined using the RELUX light simulation tool (version 2021.1.1.0) (Relux Informatik AG, Münchenstein, Switzerland) before installation (Figure 1) [22]. The confirmation of illuminance and on-site adjustments was carried out with the Extech Lux Meter EA30 (Extech Instruments Corporation, Nashua, NH, USA). The analysis of photometric properties derived from lighting laboratory photometric measurements showed that the correlated color temperature (CCT) of the luminaires ranged between 2700 kelvins (K) and 6500 K, emitting a maximum luminous flux of 10,626 lm and 11,508 lm, respectively. The wireless dimming control system enabled dimming from 100 to 0% and vice versa.

### 2.5. Activities of Daily Living Framework

Two former reports from our research group introduced an ADL framework for the fundamental assessment of near- and intermediate-vision tasks [21] and validated it in specific lighting settings (combinations of light intensity (25 fc, 50 fc, 75 fc) (1 fc (foot-candle) = 10.76 lx (lux)) and light temperature (3000 K, 4000 K, and 6000 K)) [22] (Table 1). The vision capacity of examinees for this ADL framework was measured by two general scales that standardize the participants’ score for any given ADL; the first scale uses the task duration (time required to complete a task) or a combination of task duration with the number of errors made during each task, and the second scale uses the measured number of errors.

According to the definition of the first scale, the score *s* can be calculated by the formula:

(1) $$s=\frac{1}{\ln a}\cdot \ln\frac{t}{t_{1}},\;$$

where *t*_1_ is a time parameter to be determined, and

(2) $$a=\lambda^{\frac{1}{n_{0}}},$$

where *n*_0_ is the score difference between any two times *t*_1_ and *t*_2_ whose ratio is equal to *λ* (i.e., *t*_2_ = *λ* · *t*_1_). Finally, *λ* is defined as the ratio of the average of the best 5% time over the average of the worst 5% time values required by all participants. *n*_0_ was set equal to 100.

The values of the coefficients *t*_2_, *t*_1_, *λ,* and *a* for the timescale (1) for each ADL were described extensively in our previous study [22].

The score for the second scale that assesses the number of errors *n* in ADLs is defined as follows:

(3) $$s_{n}=100e^{-bn}.$$

Parameter *b* is calculated by requiring a specific number of errors *n*_1_ to correspond to an experimentally selected score *s_n_* = 5:

(4) $$b=\frac{-\ln0.05}{n_{1}},$$

where *n*_1_ is the average number of errors of the worst 5% of performing participants.

In the present study, subtitles reading was calculated using the second scale (3), while the rest nine ADLs were calculated using the first scale (1).

### 2.6. Data Collection

All participants addressed the 10 ADLs in the following lighting settings: 1. 25 fc/3000 K, 2. 50 fc/3000 K, 3. 75 fc/3000 K, 4. 25 fc/4000 K, 5. 50 fc/4000 K, 6. 75 fc/4000 K, 7.25 fc/6000 K, 8. 50 fc/6000 K, and 9. 75 fc/6000 K. The order of the different lighting settings was randomly selected for each patient to reduce bias. A full examination in each lighting setting required an average of 10 min, including 1 min adaptation time before each light combination [27,28]. A 5 min pause was given to patients every 30 min to prevent a possible gradual decrease in concentration ability. The ADL score was calculated for all tasks based on the measured duration and/or number of errors, according to the appropriate scale defined in Equations (1) and (3). If a participant was unable to complete a task, the corresponding ADL score was 0. Apart from each ADL score, a total ADL score was calculated deriving from the mean of the 10 ADLs. The subjective preference of each participant in light combination for reading tasks was asked at the end of each examination.

The following clinical indices were evaluated: (1) binocular uncorrected near visual acuity (UNVA), (2) binocular uncorrected intermediate visual acuity (UIVA), both using the web-based Democritus Digital Acuity Reading Test (wDDART) [29,30] and binocular uncorrected distance visual acuity (UDVA) using the Greek version of ETDRS [31], and (3) spherical equivalent. Finally, the 39-item National Eye Institute Visual Functioning Questionnaire (NEI VFQ-39: NEI-VFQ-25 with some optional items) was completed by all participants [32,33].

### 2.7. Statistical Analysis

A power of 0.8 at the significance level of 0.05 for an effect size of 0.47 would be achieved using 10–16 participants in each group, according to an a priori power analysis applied for the total ADL score taking into account all lighting combinations. The normality of the measured data was evaluated using the Shapiro–Wilk test. The comparison of ADL scores for each light combination among study groups was assessed for the nine lighting settings using one-way ANOVA and the Kruskal–Wallis test for normally and non-normally distributed data, respectively. Moreover, the comparison among the nine lighting combinations for each study group for all ADL scores was performed using repeated measures ANOVA and the Friedman test for data with normal and non-normal distribution, respectively. A post hoc analysis was performed using the Tukey test and the Conover test for normally and non-normally distributed data, respectively, to address multiple comparison problems. ADL scores were correlated with age, near and total score of NEI-VFQ-39, as well as between near or total score of NEI-VFQ-39 and BUNVA, BUIVA or age using Pearson or Spearman correlation, for variables with normal and non-normal distribution, respectively. Finally, differences according to gender in ADL scores were assessed with the Mann–Whitney U test. *p*-values < 0.05 were defined as statistically significant. All statistical analyses were performed using MedCalc version 20.1.4 (MedCalc Software, Mariakerke, Belgium).

## 3. Results

The demographics of the three study groups are presented in Table 2. A total of 75 people ((25 participants in each group); 30 men (40%) and 45 women (60%)) participated in the study, with an average age of 62.65 ± 13.64 (median: 65 [57.5, 72.5]) years and the spherical equivalent of −0.57 ± 0.75 D that populated the three groups.

Significant differences were detected in UNVA (*p* < 0.001), while non-significant differences were detected in UDVA (*p* = 0.837), UIVA (*p* = 0.154), age (*p* = 0.211), and spherical equivalent (*p* = 0.120). Moreover, the total (*p* = 0.642) and near (*p* = 0.655) score of NEI-VFQ-39 also revealed non-significant differences among the three groups.

The total ADL score was significantly different among the three groups taking into account all lighting combinations, with G2 having the best total ADL score (Table 3). Moreover, taking all lighting combinations and all study groups altogether into account, ADLs were classified into three difficulty categories: (1) easy ADLs (eADLs): the three ADLs with the higher mean ADL score (reading computer screen: 98.27 ± 28.78, book reading: 97.57 ± 25.44, and subtitles reading: 89.73 ± 14.01), (2) moderate difficulty ADLs (mADLs): the four ADLs with the next higher mean ADL scores (open door test: 78.80 ± 28.56, cellular message: 71.28 ± 18.14, screwdriver test: 64.82 ± 17.21, and cellular entry search: 57.40 ± 22.16), and (3) difficult ADLs (dADLs): the three ADLs with the lower mean ADL score (supermarket receipt: 55.19 ± 14.01, drops bottle reading: 52.41 ± 26.37, and phone book search: 35.33 ± 28.79). The difference among eADLs, mADLs, and dADLs was statistically significant according to the Kruskal–Wallis test (*p* < 0.0001). Thus, the three ADL difficulty categories demonstrated sufficient construct validity since all of them could be efficiently discriminated into three distinct ADL score groups. Specifically, the median was equal to 91.38 [81.08, 110.73], 59.42 [49.17, 71.81], and 50.29 [28.56, 66.24] for eADLs, mADLs, and dADLs, respectively. In eADLs and mADLs, G2 had a significantly better total ADL score, while in dADLs, G1 appeared to have the best score (*p* < 0.0001). The comparison of ADLs separately showed significant differences among the three groups in all ADLs, with G1 having the best scores in the phone book search, drops bottle reading, cellular entry search, subtitles reading, and screwdriver tests, and with G2 having the best scores in the rest of the ADLs.

The achieved scores in each ADL were compared among the three study groups for each one of the nine combinations of light intensity and light temperature (Table 4). Significant differences were observed in the phone book search and drops bottle reading among the three study groups for all lighting combinations and in the supermarket receipt and reading computer screen tests for all lighting combinations except for the lighting combinations of 4000 K/75 fc and 4000 K/25 fc–4000 K/50 fc, respectively. On the other hand, non-significant differences were found in subtitles reading, open door test, and screwdriver test among the three study groups for all lighting combinations, as well as in the cellular message test for all lighting combinations except for the lighting combination of 3000 K/25 fc, which revealed significant difference. Book reading presented significant differences among the three study groups in the three light intensity combinations of 6000 K as well as in 3000 K/25 fc and 3000 K/50 fc, while a non-significant difference was found in the three light intensity combinations of 4000 K and in 3000 K/75 fc. Finally, the cellular entry search presented significant differences among the three study groups in the three light intensity combinations of 3000 K as well as in 4000 K/25 fc and 4000 K/75 fc, while non-significant difference was revealed in the three light intensity combinations of 6000 K and in 4000 K/50 fc.

Moreover, all ADL scores were compared among the three light intensity levels (25 fc, 50 fc, and 75 fc) for each study group and for each one of the three light temperature levels (3000 K, 4000 K, and 6000 K), as presented in Table 5. According to our results, in 4000 K, significant differences among the three light intensities were revealed only in the phone book search and the supermarket receipt test for CG and book reading for G1. Analyzing the 3000 K temperature level, G1 showed non-significant differences in all ADLs, while G2 showed significant differences among 25 fc, 50 fc, and 75 fc for the supermarket receipt, book reading, and drops bottle reading tests, and G3 for the phone book search, book reading, and screwdriver tests. Finally, regarding 6000 K, G1 showed significant differences among 25 fc, 50 fc, and 75 fc for the drops bottle reading and subtitles reading tests, G2 for the phone book search and subtitles reading tests, and G3 for the phone book search, supermarket receipt, book reading, and reading computer screen tests. 

**Figure 2:**
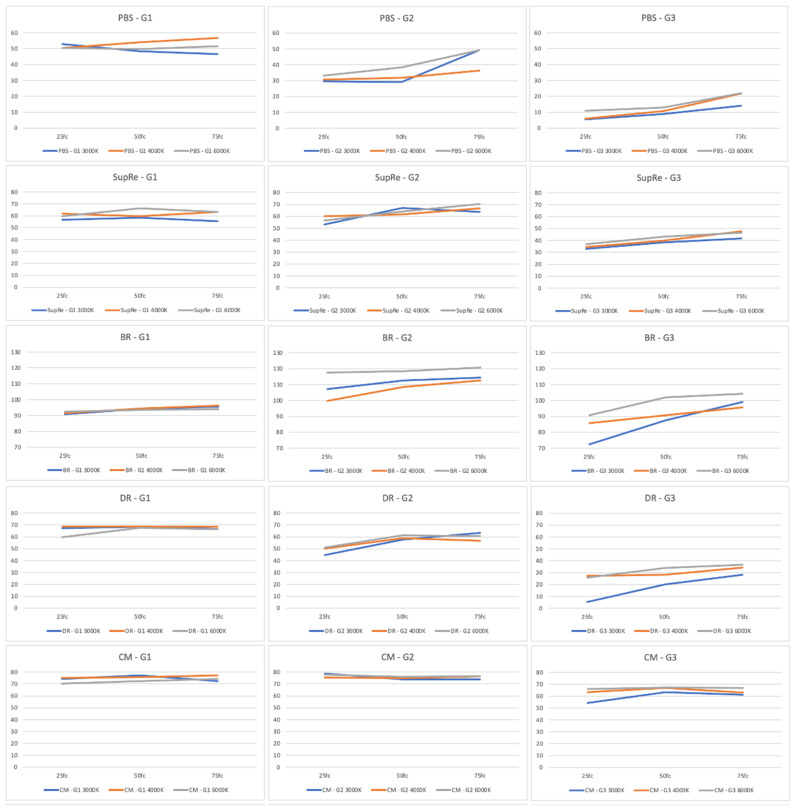

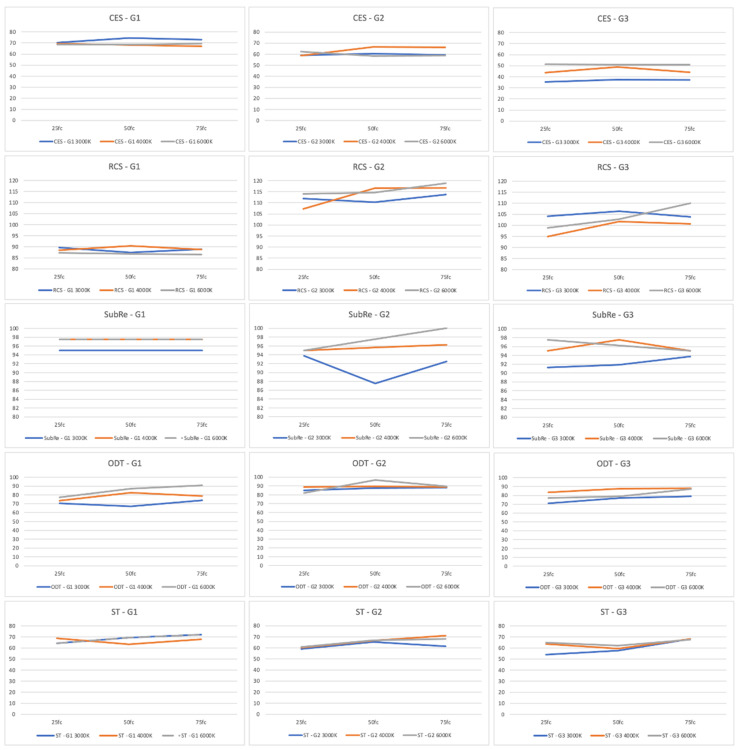
Comparison among light temperature levels (3000, 4000, and 6000 K) for the three light intensity levels (25, 50, and 75 fc) for each study group (G1: trifocal diffractive IOL, G2: bifocal hybrid IOL, G3: monofocal IOL) for all ADLs. Abbreviations: BR: book reading; CES: cellular entry search; CM: cellular message; DR: drops bottle reading; ODT: open door test; PBS: phone book search; RCS: reading computer screen; ST: screwdriver test; SubRe: subtitles reading; SupRe: supermarket receipt.

Additional comparisons were performed for all ADL scores among the three light temperature levels (3000, 4000, and 6000 K) for each study group and for the three light intensity levels (25, 50, and 75 fc), as presented in Table 5 and Figure 2. In the light intensity of 25 fc, only G3 in the book reading test presented significant differences among 3000, 4000, and 6000 K. In the light intensity of 50 fc, G2 presented non-significant differences among the three light intensity levels for all ADLs, while G1 demonstrated significant differences for the supermarket receipt, subtitles reading, and open door tests, and G3 for the book reading test. Finally, the analysis of 75 fc light intensity showed significant differences among the three light temperature levels only in G1 for the phone book search, supermarket receipt, and subtitles reading tests.

Apart from the assessment of the participants’ objective performance in all ADLs, subjective preference for lighting conditions while reading printed material was also evaluated (Table 6). Regarding light temperature, 18 participants (72%) of G1, 21 (84%) of G2, and 18 (72%) of G3 preferred 6000 K, while 7 (28%) of G1, 4 (16%) of G2, and 7 (28%) of G3 participants preferred 4000 K. No patient from all groups preferred 3000 K. Concerning light intensity, no patient from all groups preferred low light intensity (25 fc), while two participants (8%) of G1, three (12%), and zero (0%) of G3 preferred intermediate light intensity (50 fc). High light intensity (75 fc) was preferred by 23 participants (92%) of G1, 22 participants (88%) of G2, and 25 participants (100%) of G3.

Moreover, we examined which light combination showed the best total ADL, eADL, mADL, and dADL scores for each study group (Table 7, first two lines in each cell). Specifically, G1 demonstrated the best total ADL score in the light combination of 4000 K/75 fc, G2 in 6000 K/75 fc, and G3 in 6000 K/75 fc. Lighting combinations presenting the best scores in each ADL for the three study groups are also demonstrated in Table 6. All the best ADL scores were compared among the three study groups. The reading computer screen, book reading, open door test, supermarket receipt, and dADLs tests showed significantly better scores in G2; the drops bottle reading and phone book search tests showed significantly better scores in G1; while the rest of the scores showed non-significant differences among the three groups.

After finding the light combination in which each study group demonstrated the best total ADL, eADL, mADL, dADL, and each ADL score separately, as presented in two first lines of each cell in Table 7, these scores were compared with the corresponding ones in the light combination that was subjectively preferred by the majority of participants in each study group (*p*-values presented in the third line of each cell in Table 6). Namely, regarding total ADL score, G2 and G3 performed better objectively and subjectively in the same light combinations (6000 K/75 fc). On the other hand, G1 performed better objectively in 4000 K/75 fc but subjectively preferred 6000 K/75 fc as the most convenient lighting combination for reading printed material, without significant difference between these lighting combinations (total ADL score: 6000 K/75 fc: 74.76 ± 14.34, 4000 K/75 fc: 74.98 ± 14.44, *p* = 0.867). In addition, in eADLs, all groups performed better in different lighting combinations compared to their subjective preference (6000 K/75 fc); in mADLs, G2 performed better in 4000 K/75 fc, and in dADLs, G1 and G3 also performed better in 4000 K/75 fc, without significant difference compared to 6000 K/75 fc. Among all ADLs in the three groups, only G2 in the drops bottle reading test performed significantly better in 4000 K/75 fc in comparison with the subjective preference of 6000 K/75 fc.

Correlations between age and ADL scores were performed taking into consideration all lighting combinations. All groups in all ADLs showed significantly negative correlation (G1: r = −0.309 to −0.552, G2: r = −0.300 to −0.749, G3: r = −0.295 to −0.797) with *p* < 0.0001 in most cases, except for the open door test in G2, which showed no significant correlation with age (r = 0.188, *p* = 0.088).

ADL scores were compared between men and women, taking into consideration all lighting combinations. In G1, among all ADLs, the cellular message, drops bottle reading, subtitles reading, and open door Tests showed significant differences, with men having better scores than women only in the last ADL. Similarly, women in G2 had significantly better performance in all ADLs apart from the open door test, in which men performed significantly better, and in the phone book search and screwdriver test, in which no significant difference was observed. Finally, in G3, no statistical differences were detected in all ADLs except for the screwdriver test, in which men appeared to perform significantly better.

Moreover, significant correlations were observed between the total score of NEI-VFQ-39 and all ADL scores except for the eADL, book reading, and reading computer screen tests (r: 0.183 to 0.440, *p* < 0.001), while significant correlations were observed between the near score of NEI-VFQ-39 and the eADL, dADL, phone book search, book reading, reading computer screen, open door, and screwdriver tests (r: −0.178 to 0.354, *p* < 0.05). All correlations are presented in Table 8. In addition, no correlations were found between the total or near score of NEI-VFQ-39 and BUIVA, BUNVA, or age for all participants as a total, as well as for each study group (BUIVA or BUNVA with NEI-VFQ-39 (total or near score): r: −0.126 to −0.099, *p* > 0.05; age with NEI-VFQ-39 (total score): r: 0.006, *p* = 0.966; age with NEI-VFQ-39 (near score): r: 0.199, *p* = 0.174).

## 4. Discussion

It is a truism that presbyopia negatively affects the quality of life and productivity in the majority of middle-aged people due to the decrease in near-vision capacity and the high productivity requirements of working life in this age group [3,34]. Spectacles have traditionally been used as the primary conventional presbyopia correction method. However, the psychological impact of presbyopia spectacles forces many people to avoid near-vision activities in order not to use their spectacles. Indeed, it is estimated that the average American citizen is willing to pay at least USD 5 per day to be spectacle independent for his/her near-vision activities [35]. A variety of surgical therapeutic options are available, with premium IOL implantations being an increasingly common presbyopia correction method [11,12,13]. However, despite the impressive progress, presbyopia is yet to be fully addressed.

Clinical experience suggests that reading ability and general visual performance depend highly on light conditions [36]. Therefore, presbyopia is a multifactorial pathological entity that is highly dependent on environmental lighting conditions. Lighting societies attempt to address lighting needs by introducing lighting directives for both indoor and outdoor settings. However, these directives are primarily based on subjective reports from research subjects and phasmatoscopic examinations using enucleated eyes rather than on actual experimental data regarding visual performance in the proposed settings [37,38]. Additionally, no lighting directives have been issued for pseudophakic patients implanted with monofocal or multifocal IOLs. Finally, since lighting needs may differ according to the task being completed, different lighting guidelines should be available for each daily activity, so that lighting settings result in neither suboptimal visual capacity nor energy pollution and waste [38,39].

Finally, the retinal illuminance, namely the luminous flux incident on the retina, depends not only on the external stimulus brightness but also on the age, lens clarity, and pupil diameter [40,41,42]. For instance, the retinal illuminance of a 60-year-old adult is approximately one-third of the corresponding value of a 20-year-old adult due to the decrease in the pupil diameter and the yellowing of the lens with age [41]. Similarly, we can suppose that retinal illuminance can also depend on the IOL type implanted in the eye after cataract extraction surgery. Since monofocal, bifocal, and trifocal IOLs split the light beam into one, two, or three parts, respectively, we can understand why multifocal IOLs deliver a lower percentage of the light to the near and/or intermediate focal point in comparison to a monofocal IOL or crystalline lens.

Taking everything mentioned into consideration, the question arises as to whether light temperature, light intensity, and IOL type affect the visual performance of ADLs requiring near and intermediate visual capacity. Within this context, the primary objective of the present study was to objectively compare the visual performance of patients implanted binocularly with trifocal diffractive, bifocal hybrid, or monofocal IOLs in an experimental lighting facility in different user-defined combinations of light intensity and light temperature for series of ADLs that require near and intermediate vision. Moreover, among the objectives of this study was to reveal potential differences that could serve as the scientific background for the development of lighting recommendations to these groups of patients that would ensure optimal visual capacity without energy pollution and waste.

To address these objectives, a hospital ward was transformed into an already validated high-end artificial lighting facility that provides uniform illuminance conditions both in terms of light intensity and light temperature [22]. Participants were divided equally into three groups according to the type of IOL implanted after cataract surgery: a group with bilateral implantation of the trifocal IOL Panoptix, a group with bilateral implantation of the bifocal IOL Restor, and a group with bilateral implantation of the monofocal IOL SF60WF. All participants had to address a series of ADLs that simulate common daily tasks in nine combinations of light intensity and light temperature. Performance scoring in each ADL was conducted using two nonlinear mathematical models that evaluate time duration and potential errors.

For better and easier evaluation of our results, ADLs were divided into three groups according to their difficulty, and the corresponding scores were calculated for each participant: easy ADL score (eADL score), moderate ADL score (mADL score), and difficult ADL score (dADL score). eADLs and mADLs do not have such high demands on good near vision, while dADLs need a very good near vision capacity to be successfully performed. A total ADL score was also calculated for each participant. Taking all lighting combinations into account, patients implanted bilaterally with Restor IOLs (G2) had the best total ADL score among the three IOL groups. Similarly, participants of G2 performed better in eADLs and mADLs, while participants of G1 performed better in dADLs. The better performance of G1 in dADLs could be explained by the significantly better BUNVA of G1. The better performance of the trifocal Panoptix at a near distance is explained by the higher modulation transfer function (MTF) values in comparison to the bifocal Restor +2.5 D and the monofocal AcrySof IQ SN60WF [43]. Similarly, the better performance of G2 in eADLs and mADLs could be explained by the better, even at no significant level, BUIVA of G2 in comparison to G1 and G3. The better performance of Restor +2.5 D at an intermediate distance was confirmed by Cardona et al. [44], who found that the trifocal diffractive IOL AT LISA tri (+3.33 D near and +1.66 D intermediate add power) did not perform better than Restor +2.5 at 60 cm, and by Carson et al. [45], who found better resolution with fewer background shadows at 50 and 60 cm with Restor +2.5 D in comparison with two aspheric trifocal diffractive IOLs (AT LISA tri 839MP) and FineVision Micro F12 trifocal IOLs (+3.5 D near and +1.75 D intermediate add power)).

When ADL scores were compared among the three study groups for each light combination, in the two most difficult ADLs, phone book search and drops bottle reading, both using printed material, G1 had significantly better scores in all light combinations. On the other hand, in the supermarket receipt test, which was the third most difficult ADL using printed material, G2 performed significantly better than G1 in the majority of lighting combinations, while concerning book reading, which is considered an easy ADL using printed material, G2 also performed better than G1 in all 6000 K combinations, in 3000 K/25 fc, and in 3000 K/50 fc. These results were compared with another study of our research group examining reading capacity with the printed Greek version of the MNREAD acuity chart (MNREAD-GR) at 40 cm using task lighting in the same nine combinations of light intensity and light temperature among patients implanted bilaterally with Panoptix, Restor, or SF60WF IOLs [46]. According to this study, the Panoptix group had better BUNVA than the rest of the study participants at all light intensities (all *p* < 0.01), followed by the Restor group, while significantly worse reading capacity at all light conditions was detected in the monofocal group. A minimal reading speed of 80 words per minute was a prerequisite, without taking the reading time into consideration for the final assessment of patients’ performance, in contrast to the present study, in which all ADL scores took the task duration into account.

Regarding ADLs using digital screens, when compared among the three study groups for each light combination, reading from a computer screen, the easiest ADL, showed a better score in G2 in all lighting combinations appearing with statistical significance in all light combinations apart from 4000 K/25 fc and 4000 K/50 fc. Finally, the cellular message, subtitles reading, open door, and screwdriver tests showed non-significant differences among the three groups in all or in the majority of lighting combinations.

When ADL scores were compared among the three light intensity levels (25, 50, and 75 fc) for each study group and each light temperature level, we found that the Panoptix group presented significant differences in only 3 (one in 4000 K and two in 6000 K) of the 30 cases (10 ADLs × 3 light temperature levels), the Restor group in 5 cases (three in 3000 K and two in 6000 K), and the monofocal group in 9 cases (three in 3000 K, two in 4000 K, and four in 6000 K). Therefore, it seems that Panoptix IOL is the most light intensity-independent group since only in three cases (6000 K-drops bottle reading, 6000 K-subtitles reading, and 4000 K-book reading) did the ADL score differentiate significantly among the three light intensity levels, which is compatible with the results of our previous study [46]. On the other hand, the monofocal group was the most light intensity-dependent group.

ADL scores were also compared among the three light temperature levels (3000 K, 4000 K, and 6000 K) for each study group and each light intensity level. The Panoptix group presented significant differences in six cases (three in 50 fc and three in 75 fc), the Restor group in no case, and the monofocal group in two cases (one in 25 fc and one in 50 fc) among the 30 cases (10 ADLs × 3 light intensity levels). These findings indicate that the Restor group is light temperature-independent, while Panoptix IOL is the most light temperature-dependent group. These outcomes cannot be directly compared with our previous study [45] since no comparison was performed among the light temperature levels in the examined IOL groups.

Finally, the light combination in which each study group presented the best scores was evaluated for each ADL and for total ADL, eADL, mADL, and dADL scores. The Restor and monofocal groups had the best total ADL scores in cold and intense light (6000 K/75 fc), while, interestingly, the Panoptix group had the best total ADL score in moderate light temperature (daylight) and in high light intensity (4000 K/75 fc). Our results are similar to the outcomes of our previous study [45], in which the monofocal group had the best reading capacity in 6000 K/75 fc, the Restor group had an almost flawless reading performance in 6000 K/75 fc with similar results in 6000/50 fc and 4000/75 fc, and the Panoptix group had the best reading performance in 4000 K/75 fc and a non-significant light intensity-independent reduction in 6000 K.

Apart from the objective assessment of patients’ capacity in all ADLs, each participant had to declare the optimal light combination while reading that was the most comfortable according to his/her subjective opinion. No group selected low light temperature (3000 K) as the most comfortable combination. Similarly, no group selected low light intensity (25 fc) from each light temperature. These findings are compatible with the previous corresponding study of our group with the exception of two patients implanted with Restor IOL who selected 4000 K/25 fc as the most comfortable combination while reading printed material [46]. Moreover, 72% (18/25) of the Panoptix and monofocal group selected cold light (6000 K), and 28% (7/25) of the same groups selected daylight (4000 K) as the most comfortable lighting temperature, while 84% (21/25) of the Restor group selected 6000 K and 16% (4/25) 4000 K. On the other hand, in our previous study [46], the majority (68%) of the Panoptix and Restor groups preferred 4000 K, while 100% of the monofocal group selected 6000 K. Additionally, in our present study, 92% (23/25) of the Panoptix group, 88% (22/25) of the Restor group, and 100% (25/25) of the monofocal group preferred high (75 fc) light intensity, while in our previous study, 44% (11/25) of the Panoptix group, 88% (22/25) of the Restor group, and 92% (23/25) of the monofocal group preferred 75 fc. Correspondingly, in our present study, 8% (2/25) of the Panoptix group, 12% (3/25) of the Restor group, and 0% (0/25) of the monofocal group selected intermediate (50 fc) light intensity, while in our previous study 48% (12/25) of the Panoptix group, 12% (3/25) of the Restor group, and 8% (2/25) of the monofocal group preferred 50 fc. These differences may be attributed to the fact that in the present study, the lighting was ambient, while in our previous study, task lighting was used.

After finding the light combined with the best ADL scores and the most comfortable light combination for reading of each study group, we compared them in order to find if subjective preference in light coincides with the objective performance of each group. Among the three study groups, the Restor and monofocal groups performed better (total ADL score) in 6000 K/75 fc and simultaneously preferred the same light combination as the most comfortable, while Panoptix patients performed better in 4000 K/75 fc, although they preferred 6000 K/75 fc as the most comfortable light combination. However, all ADL scores of the Panoptix group were compared between 6000 K/75 fc and 4000 K/75 fc, and no significant difference was observed. Therefore, even if the patients of this group preferred reading in 6000 K/75 fc, their reading performance was not reduced in this light condition in comparison with the light condition in which they performed optimally.

Finally, some correlations and comparisons of ADL scores were performed with demographic and clinical parameters for the best possible evaluation of our outcomes. First, an expected finding was the significantly negative correlation between the vast majority of ADL scores and age in all study groups, meaning that the older the patients, the lower their ADL scores. Additionally, ADL scores were found to be influenced by gender, mainly in Restor patients and hardly at all in monofocal patients. Generally, in the majority of ADLs, especially in those that require reading fluency, women had significantly better scores, while in ADLs requiring manual dexterity, such as the open door test and screwdriver test, men performed significantly better. However, it should be taken into account that the monofocal group had an almost equal number of men and women, while in the Panoptix and Restor groups, women outnumbered men insignificantly.

To our knowledge, this is the first study that explores the impact of lighting conditions on the objective visual capacity of patients implanted bilaterally with trifocal diffractive, bifocal hybrid, or monofocal IOLs in an experimental lighting facility which allows the simulation of a series of ADLs. These lighting combinations reflect different illuminance scenarios that are commonly encountered in public, home, and work settings. However, certain limitations of the study should be noted prior to the interpretation of our results. First, no randomization was possible among the multifocal groups since multifocal IOL selection depends not only on clinical criteria but also on lifestyle, personality, work, and other personal characteristics. Moreover, the subjective preference of patients in light intensity and light temperature combinations was evaluated only for reading and not for each ADL separately. Therefore, conclusions can be reached safely only for ADLs, including reading on printed material, such as the phone book search, supermarket receipt, book reading, and drops bottle reading tests. In future studies, subjective preference in lighting conditions could be evaluated not only during reading on printed material but also during activities requiring manual dexterity and usage of digital screens such as mobile phone and computer screens. In addition, ADLs in the present study were grouped into three IOL groups according to their difficulty to be performed. In future studies, ADLs could also be grouped according to the type of the ADL, such as ADLs evaluating reading in printed material, ADLs evaluating reading in digital screens, or ADLs requiring manual dexterity for the most comprehensive assessment possible and the best possible understanding of the results. Indeed, further studies with larger cohorts of patients are necessary to confirm our outcomes. Moreover, further to the daily tasks requiring near and intermediate vision, evaluation of other activities of daily living requiring distance vision in predefined lighting will help us to identify the optimal light conditions for working or home environments that will improve multifocal IOL performance. Finally, this validated lighting setting could also be applied in patients with low vision, such as patients with diabetic retinopathy, age-related macular edema, glaucoma, or other ocular diseases.

## 5. Conclusions

To summarize, monofocal patients may be spectacle-independent in easy and moderate difficulty daily activities, especially when intense, cold lighting is available. Bifocal patients also benefit from intense, cold lighting, and, in the majority of their near- and intermediate-vision tasks, they would be spectacle-free since they present superiority in daily tasks of low and moderate difficulty. Finally, trifocal patients benefit from intense daylight instead of the prevalent intense, cold lighting of modern working settings and present superior near vision capacity in difficult near-vision daily tasks in comparison with bifocal and monofocal patients. Generally, patients implanted with bifocal hybrid IOLs are more light temperature-independent, while patients implanted with trifocal diffractive IOLs are more light intensity-independent and more light temperature-dependent.

In conclusion, near- and intermediate-vision capacity depends heavily on light conditions. Lighting conditions do actually improve or worsen the performance of patients implanted with multifocal IOLs. It becomes obvious that the actual visual performance of these patients should rely on objective measurement of their capacity to address common daily tasks for the best possible assessment of the IOL-type impact on the patient’s performance. Moreover, this approach will allow the introduction of updated lighting norms for public and private settings that will address the special lighting needs of multifocal pseudophakic patients without resulting in energy waste and pollution.

## Figures and Tables

**Table 1:** Detailed description of activities of daily living (ADLs) [22].

ADL	Description	Measured Parameter
Phone Book Search (PBS)	Patient is required to find and read a specific entry in a regular phonebook catalog in the nine different lighting settings. A different entry is asked to be found in each combination.	task duration
Supermarket Receipt (SupRe)	Patient is required to find and read three products from a typical supermarket receipt (monospaced Sanserif font) in the nine different lighting settings. Different products are asked to be found in each combination.	task duration + (number of errors × 2)
Book Reading (BR)	Patient is required to read an excerpt of a predetermined length in a novel in the nine different lighting settings. A different excerpt of the same length is asked to be read in each combination.	task duration + (number of errors × 2)
Drops bottle Reading (DR)	Patient is required to correctly read the expiration date printed on three typical eye dropper bottles in the nine different lighting settings. Different eye dropper bottles are used in each combination.	task duration + (number of errors × 2)
Cellular Message (CM)	Patient is required to read a Short Message Service (SMS) on a 5-inch cellular phone ^a^ in the nine different lighting settings. A different SMS is asked to be read in each combination.	task duration + (number of errors × 2)
Cellular Entry Search (CES)	Patient is required to find and read one specific entry on a 5-inch cellular phone ^a^ in the nine different lighting settings. Different entries are asked to be found in each combination.	task duration
Reading Computer Screen (RCS)	Patient is required to correctly read the text of a predetermined length from a computer screen ^b^ in the nine different lighting settings. Different texts of the same length are asked to be read in each combination.	task duration + (number of errors × 2)
Subtitles Reading (SubRe)	Patient is required to correctly read movie subtitles from a one-minute movie clip on a computer screen ^b^ in the nine different lighting settings. Different movie clips are shown in each combination.	number of errors
Open Door Test (ODT)	Patient is required to find a specific key from a keychain that holds 10 keys and insert it in the corresponding door keyhole in the nine different lighting settings. Different keychains are given in each combination.	task duration
Screwdriver Test (ST)	Patient is required to select one among three screwdrivers and insert it in the appropriate screw among a variety of screw types in the nine different lighting settings. Different screwdrivers are given to be selected in each combination.	task duration

Task duration (in seconds); ^a^ Cellular screen specifications: screen resolution 720 × 1280 pixels; pixel density ~294 ppi; screen brightness 100%; ^b^ Computer screen specifications: 13.3-inch screen; screen resolution 3840 × 2160 pixels; pixel density 331.3 ppi; Times New Roman font size 12; zoom 150%; brightness 100%; contrast 50%.

**Table 2:** Demographic and clinical parameters of study groups.

Parameters	G1	G2	G3	*p*-Value
	Mean ± SD Median (Interquartile Range)			
N	25	25	25	NA
Gender (Male/Female)	9/16	9/16	12/13	0.249
Age (years)	60.23 ± 13.54	60.75 ± 16.26	68.07 ± 24.45	0.211
Spherical equivalent (D)	−0.37 ± 0.65	−0.74 ± 1.04	−0.77 ± 0.60	0.120
BUDVA (logMAR)	0.00 [−0.05, 0.10]	−0.02 [−0.06, 0.04]	0.00 [−0.10, −0.11]	0.837
BUIVA (logMAR)	−0.02 [−0.08, 0.02]	−0.06 [−0.14, −0.01]	−0.04 [−0.06, −0.14]	0.154
BUNVA (logMAR)	0.10 [0.00, 0.10]	0.13 [0.05, 0.30]	0.30 [0.25, 0.35]	<0.001 *
NEI-VFQ−39 (total score)	88.52 ± 8.67	84.15 ± 20.74	87.20 ± 23.39	0.642
NEI-VFQ−39 (near score)	83.14 ± 13.70	77.78 ± 22.91	87.20 ± 27.90	0.355

* *p* < 0.001. Abbreviations: D: Diopters; NA: not applicable; BUDVA: binocular uncorrected distance visual acuity; BUIVA: binocular uncorrected intermediate visual acuity; BUNVA: binocular uncorrected near visual acuity; G1: trifocal group; G2: bifocal group; G3: monofocal group; SD: standard deviation.

**Table 3:** Comparisons among the 3 groups taking into account all light combinations.

Scores	G1	G2	G3	*p*-Value (Conover Test)
	Median (Interquartile Range)			
Total score	74.35 [65.14, 81.89]	**78.17 [68.07, 83.49]**	58.62 [48.41, 70.09]	**<0.001** ** (G1-G3, G2-G3)
Easy ADL score	89.37 [81.72, 103.27]	**110.07 [90.97, 120.11]**	88.95 [74.78, 105.37]	**<0.001** ** (G1-G2, G2-G3)
Moderate ADL score	58.29 [51.16, 69.33]	**71.65 [58.25, 77.30]**	53.95 [44.23, 63.27]	**<0.001** ** (G1-G2, G2-G3, G1-G3)
Difficult ADL score	**60.74 [47.46, 71.63]**	56.41 [32.56, 62.45]	23.81 [14.29, 36.61]	**<0.001** ** (G1-G2, G2-G3, G1-G3)
RCS (eADL)	83.71 [73.06, 105.58]	**116.64 [92.13, 130.43]**	108.01 [83.72, 122.49]	**<0.001** ** (G1-G2, G2-G3, G1-G3)
BR (eADL)	88.61 [80.29, 112.42]	**119.46 [94.89, 132.27]**	92.11 [76.00, 109.54]	**<0.001** ** (G1-G2, G2-G3)
SubRe (eADL)	**97.50 [89.69, 100.00]**	95.00 [82.82, 100.00]	94.38 [78.75, 100.00]	**0.044** * (G1-G3)
ODT (mADL)	74.15 [55.87, 95.06]	**87.42 [68.67, 107.25]**	76.60 [61.40, 93.58]	**0.014** * (G1-G2, G2-G3)
CM (mADL)	76.52 [63.67, 86,28]	**79.91 [62.88, 89.05]**	65.08 [49.96, 74.22]	**<0.001** ** (G1-G3, G2-G3)
ST (mADL)	**69.94 [55.42, 78.93]**	64.06 [54.09, 74.25]	61.28 [53.07, 71.60]	**0.036** * (G1-G3)
CES (mADL)	**69.15 [51.59, 78.05]**	65.01 [53.13, 72.59]	44.13 [24.51, 60.33]	**<0.001** ** (G1-G3, G2-G3)
SupRe (dADL)	61.29 [50.26, 73.87]	**63.43 [48.36, 77.29]**	42.99 [22.51, 56.26]	**<0.001** ** (G1-G3, G2-G3)
DR (dADL)	**66.86 [51.97, 106.56]**	60.34 [44.61, 103.18]	26.37 [9.43, 70.08]	**<0.001** ** (G1-G2, G2-G3, G1-G3)
PBS (dADL)	**52.54 [25.97, 67.47]**	35.98 [2.77, 51.26]	0.00 [0.00, 19.77]	**<0.001** ** (G1-G2, G2-G3, G1-G3)

* *p* < 0.05, ** *p* < 0.000001, in bold: the highest ADL scores among the three study groups. Abbreviations: ADLs: activities of daily living; BR: book reading; CES: cellular entry search; CM: cellular message; dADLs: difficult ADLs; DR: drops bottle reading; eADLs: easy ADLs; G1: trifocal group, G2: bifocal group, G3: monofocal group; mADLs: moderate difficulty ADLs; ODT: open door test; PBS: phone book search; RCS: reading computer screen; ST: screwdriver test; SubRe: subtitles reading; SupRe: supermarket receipt.

**Table 4:** Comparisons among the study groups for all lighting combinations.

ADLs	Lighting Settings	G1	G2	G3	*p*-Value (^a^ One-Way ANOVA/ ^b^ Kruskal–Wallis Test) (^a^ Tukey Test/ ^b^ Conover Test)
		Mean ± SD Median (Interquartile Range)			
PBS	3000 K/25 fc	**52.82 ± 27.46**	29.50 ± 29.03	7.33 ± 15.35	**<0.001** ^a,#^ (G1-G2, G1-G3)
	3000 K/50 fc	**45.99 ± 24.20**	29.01 ± 26.75	8.97 ± 17.02	**<0.001** ^a,#^ (G1-G3)
	3000 K/75 fc	**46.57 ± 20.93**	40.22 ± 30.82	13.30 ± 19.89	**0.001** ^a,^** (G1-G3, G2-G3)
	4000 K/25 fc	**50.42 ± 25.02**	30.59 ± 23.59	5.88 ± 17.99	**<0.001** ^a,#^ (G1-G3, G2-G3)
	4000 K/50 fc	**54.11 ± 22.61**	31.92 ± 23.85	10.77 ± 21.35	**<0.001** ^a,#^ (G1-G2, G1-G3)
	4000 K/75 fc	**56.70 ± 22.62**	36.38 ± 25.83	21.74 ± 29.03	**0.002** ^a,^** (G1-G3)
	6000 K/25 fc	**49.55 ± 23.10**	30.21 ± 27.02	10.99 ± 16.17	**<0.001** ^a,#^ (G1-G3)
	6000 K/50 fc	**48.46 ± 18.30**	38.29 ± 31.40	11.96 ± 17.93	**<0.001** ^a,#^ (G1-G3, G2-G3)
	6000 K/75 fc	**51.62 ± 22.92**	44.78 ± 41.56	20.22 ± 25.24	**0.014** ^a,^* (G1-G3)
SupRe	3000 K/25 fc	**56.63 ± 23.71**	52.19 ± 23.39	33.70 ± 19.14	**0.016** ^a,^* (G1-G3)
	3000 K/50 fc	57.35 ± 23.15	**66.93 ± 22.77**	39.12 ± 19.26	**0.012** ^a,^* (G2-G3)
	3000 K/75 fc	55.53 ± 20.09	**63.62 ± 24.06**	41.66 ± 19.68	**0.039** ^a,^* (G2-G3)
	4000 K/25 fc	**61.69 ± 20.58**	59.99 ± 26.05	33.05 ± 27.02	**0.004** ^a,^** (G1-G3, G2-G3)
	4000 K/50 fc	59.86 ± 18.05	**61.77 ± 15.16**	36.07 ± 22.93	**0.002** ^a,^** (G1-G3, G2-G3)
	4000 K/75 fc	64.31 ± 23.71	**66.73 ± 20.67**	47.58 ± 18.38	0.069 ^a^
	6000 K/25 fc	**59.76 ± 18.15**	58.56 ± 18.87	36.94 ± 25.90	**0.009** ^a,^** (G1-G3, G2-G3)
	6000 K/50 fc	**66.09 ± 22.15**	64.05 ± 15.07	43.33 ± 20.81	**0.009** ^a,^** (G1-G3, G2-G3)
	6000 K/75 fc	63.41 ± 23.06	**71.34 ± 18.05**	44.79 ± 19.98	**0.008** ^a,^** (G1-G3, G2-G3)
BR	3000 K/25 fc	90.89 ± 23.49	**107.09 ± 23.76**	75.31 ± 32.89	**0.016** ^a,^* (G2-G3)
	3000 K/50 fc	92.50 ± 24.11	**112.71 ± 23.67**	87.44 ± 21.94	**0.025** ^a,^* (G2-G3)
	3000 K/75 fc	95.52 ± 25.53	**114.47 ± 24.73**	102.10 ± 21.84	0.119 ^a^
	4000 K/25 fc	91.34 ± 23.07	**99.74 ± 32.37**	84.35 ±32.29	0.438 ^a^
	4000 K/50 fc	94.56 ± 21.60	**108.48 ± 26.22**	89.34 ± 25.24	0.161 ^a^
	4000 K/75 fc	96.17 ± 22.00	**112.62 ± 27.06**	95.70 ± 26.29	0.186 ^a^
	6000 K/25 fc	91.59 ± 21.57	**113.36 ± 25.11**	90.79 ± 26.43	**0.031** ^a,^* (G1-G2)
	6000 K/50 fc	92.49 ± 21.41	**118.52 ± 21.60**	102.02 ± 19.43	**0.007** ^a,^** (G1-G2)
	6000 K/75 fc	94.09 ± 22.14	**116.78 ± 20.97**	102.19 ± 18.91	**0.018** ^a,^* (G1-G2)
DR	3000 K/25 fc	**67.19 [43.90, 77.41]**	45.34 [29.68, 62.57]	5.32 [2.12, 34.36]	**<0.001** ^b,#^ (G1-G3, G2-G3)
	3000 K/50 fc	**68.51 [51.80, 84.51]**	65.03 [40.89, 71.27]	20.19 [8.80, 40.47]	**<0.001** ^b,#^ (G1-G3, G2-G3)
	3000 K/75 fc	**66.79 [55.16, 79.60]**	64.27 [52.52, 78.06]	29.40 [20.63, 49.10]	**<0.001** ^b,#^ (G1-G3, G2-G3)
	4000 K/25 fc	**66.88 [51.70, 80.64]**	56.28 [47.30, 68.80]	14.86 [7.94, 48.93]	**<0.001** ^b,#^ (G1-G3, G2-G3)
	4000 K/50 fc	**68.90 [55.22, 82.32]**	66.04 [50.92, 76.30]	27.24 [8.11, 38.31]	**<0.001** ^b,#^ (G1-G3, G2-G3)
	4000 K/75 fc	**68.56 [61.55, 79.45]**	68.18 [42.08, 74.15]	39.17 [17.97, 53.65]	**0.001** ^b,**^ (G1-G3, G2-G3)
	6000 K/25 fc	**59.32 [44.18, 66.58]**	60.78 [35.45, 71.43]	25.66 [5.33, 44.35]	**0.003** ^b,**^ (G1-G3, G2-G3)
	6000 K/50 fc	**67.18 [57.86, 77.67]**	63.10 [49.55, 72.21]	33.05 [22.25, 44.95]	**<0.001** ^b,#^ (G1-G3, G2-G3)
	6000 K/75 fc	**66.40 [53.16, 86.14]**	59.80 [49.24, 81.57]	29.09 [11.06, 54.45]	**0.002** ^b,**^ (G1-G3, G2-G3)
CM	3000 K/25 fc	74.21 ± 14.63	**78.83 ± 16.05**	53.85 ± 16.83	**<0.001** ^a,#^ (G1-G3, G2-G3)
	3000 K/50 fc	76.46 ± 15.51	73.98 ± 16.76	63.42 ± 20.15	0.119 ^a^
	3000 K/75 fc	72.24 ± 17.96	**73.91 ± 16.80**	63.15 ± 20.75	0.285 ^a^
	4000 K/25 fc	73.28 ± 17.01	**75.32 ± 18.77**	61.73 ± 18.72	0.141 ^a^
	4000 K/50 fc	**75.74 ± 13.92**	74.74 ± 12.03	64.88 ± 17.85	0.125 ^a^
	4000 K/75 fc	**77.07 ± 13.94**	76.17 ± 16.64	62.92 ± 20.67	0.072 ^a^
	6000 K/25 fc	70.77 ± 19.87	**75.55 ± 19.62**	66.01 ± 13.63	0.454 ^a^
	6000 K/50 fc	72.77 ± 18.46	**76.12 ± 18.45**	67.26 ± 13.24	0.460 ^a^
	6000 K/75 fc	74.23 ± 22.08	**74.77 ± 23.14**	64.09 ± 21.52	0.371 ^a^
CES	3000 K/25 fc	**63.57 ± 22.26**	58.80 ± 16.96	37.69 ± 23.36	**0.004** ^a,^** (G1-G3, G2-G3)
	3000 K/50 fc	**64.60 ± 23.90**	60.37 ± 14.09	37.47 ± 24.31	**0.006** ^a,^** (G1-G3, G2-G3)
	3000 K/75 fc	**65.70 ± 22.78**	59.28 ± 12.53	38.96 ± 23.43	**0.003** ^a,^** (G1-G3, G2-G3)
	4000 K/25 fc	**63.50 ± 22.16**	60.07 ± 16.13	40.65 ± 23.35	**0.016** ^a,^* (G1-G3)
	4000 K/50 fc	62.07 ± 17.43	**63.79 ± 14.04**	45.75 ± 24.15	**0.042** ^a,^* (G2-G3)
	4000 K/75 fc	**63.12 ± 20.01**	62.51 ± 16.48	43.93 ± 23.91	**0.040** ^a,^* (G1-G3)
	6000 K/25 fc	**65.11 ± 24.27**	64.22 ± 20.49	51.36 ± 20.55	0.787 ^a^
	6000 K/50 fc	**61.65 ± 22.09**	58.39 ± 19.05	50.85 ± 17.35	0.343 ^a^
	6000 K/75 fc	**65.14 ± 21.67**	59.65 ± 21.02	49.52 ± 19.69	0.124 ^a^
RCS	3000 K/25 fc	89.63 ± 24.19	**112.01 ± 23.05**	104.26 ± 22.46	**0.033** ^a,^* (G1-G2)
	3000 K/50 fc	85.74 ± 25.44	**110.24 ± 22.68**	106.37 ± 23.39	**0.013** ^a,^* (G1-G2)
	3000 K/75 fc	88.91 ± 27.97	**113.73 ± 24.85**	105.50 ± 28.23	**0.045** ^a,^* (G1-G2)
	4000 K/25 fc	88.25 ± 26.15	**107.28 ± 41.63**	92.30 ± 38.36	0.344 ^a^
	4000 K/50 fc	90.43 ± 26.27	**116.58 ± 49.51**	98.44 ± 28.17	0.145 ^a^
	4000 K/75 fc	88.86 ± 29.17	**116.72 ± 20.38**	100.70 ± 26.36	**0.034** ^a,^* (G1-G2)
	6000 K/25 fc	86.19 ± 25.98	**111.78 ± 22.37**	98.77 ± 26.48	**0.026** ^a,^* (G1-G2)
	6000 K/50 fc	85.28 ± 25.65	**114.66 ± 21.97**	102.78 ± 26.60	**0.009** ^a,^** (G1-G2)
	6000 K/75 fc	86.46 ± 26.61	**115.93 ± 23.15**	106.86 ± 31.12	**0.012** ^a,^* (G1-G2)
SubRe	3000 K/25 fc	**95.00 [85.63, 99.38]**	93.75 [81.25, 100.00]	90.00 [78.13, 98.13]	0.665 ^b^
	3000 K/50 fc	**93.75 [88.13, 97.50]**	87.50 [80.21, 97.50]	91.88 [82.50, 98.13]	0.718 ^b^
	3000 K/75 fc	**95.00 [92.50, 97.50]**	92.50 [81.46, 96.25]	93.75 [86.25, 98.75]	0.599 ^b^
	4000 K/25 fc	**97.50 [85.00, 100.00]**	95.00 [85.00, 100.00]	95.00 [63.13, 97.50]	0.307 ^b^
	4000 K/50 fc	**97.50 [88.75, 100.00]**	95.63 [87.50, 100.00]	97.50 [71.88, 100.00]	0.634 ^b^
	4000 K/75 fc	**97.50 [95.00, 100.00]**	96.25 [72.50, 100.00]	95.00 [77.50, 100.00]	0.710 ^b^
	6000 K/25 fc	**97.50 [83.13, 100.00]**	95.00 [81.25, 100.00]	97.50 [78.13, 100.00]	0.847 ^b^
	6000 K/50 fc	**97.50 [89.38, 100.00]**	97.50 [89.06, 100.00]	96.88 [76.25, 100.00]	0.937 ^b^
	6000 K/75 fc	97.50 [95.00, 100.00]	**100.00 [83.75, 100.00]**	95.00 [83.75, 98.75]	0.278 ^b^
ODT	3000 K/25 fc	70.43 ± 30.24	**84.92 ± 23.68**	71.00 ± 19.90	0.381 ^a^
	3000 K/50 fc	67.32 ± 40.36	**87.81 ± 29.56**	76.82 ± 21.99	0.376 ^a^
	3000 K/75 fc	73.76 ± 36.01	**79.23 ± 40.84**	79.07 ± 19.06	0.301 ^a^
	4000 K/25 fc	69.52 ± 34.07	**79.17 ± 27.60**	68.79 ± 22.50	0.686 ^a^
	4000 K/50 fc	82.47 ± 28.45	**92.38 ± 24.02**	73.65 ± 23.13	0.294 ^a^
	4000 K/75 fc	78.86 ± 31.68	**92.05 ± 25.69**	77.78 ± 27.28	0.491 ^a^
	6000 K/25 fc	65.35 ± 34.15	**80.52 ± 25.54**	77.04 ± 25.13	0.442 ^a^
	6000 K/50 fc	79.91 ± 38.27	**96.63 ± 30.77**	79.03 ± 13.19	0.329 ^a^
	6000 K/75 fc	**90.78 ± 19.33**	84.07 ± 36.35	87.12 ± 17.09	0.844 ^a^
ST	3000 K/25 fc	**61.89 ± 21.26**	58.88 ± 12.81	54.53 ± 21.36	0.582 ^a^
	3000 K/50 fc	62.61 ± 16.82	**65.46 ± 13.99**	57.69 ± 24.35	0.597 ^a^
	3000 K/75 fc	**69.89 ± 15.53**	61.33 ± 17.00	68.32 ± 15.58	0.360 ^a^
	4000 K/25 fc	**69.42 ± 18.97**	60.14 ±21.14	62.37 ± 21.16	0.432 ^a^
	4000 K/50 fc	63.40 ± 17.05	**66.64 ± 16.49**	58.60 ± 14.21	0.535 ^a^
	4000 K/75 fc	67.82 ± 18.70	**70.98 ± 10.48**	68.05 ± 13.55	0.879 ^a^
	6000 K/25 fc	63.94 ± 17.69	59.97 ± 15.52	**64.85 ± 17.98**	0.757 ^a^
	6000 K/50 fc	**68.58 ± 13.13**	66.72 ± 12.46	62.18 ± 13.84	0.426 ^a^
	6000 K/75 fc	**72.20 ± 18.19**	66.97 ± 13.90	65.21 ± 19.65	0.500 ^a^

* *p* < 0.05, ** *p* < 0.01, ^#^ *p* < 0.001 (in bold); ^a^ One-Way ANOVA & Tukey Test/^b^ Kruskal–Wallis Test & Conover Test; in bold: the highest ADL scores among the three study groups for each light combination. Abbreviations: ADLs: activities of daily living, BR: book reading; CES: cellular entry search; CM: cellular message; DR: drops bottle reading; G1: trifocal group, G2: bifocal group, G3: monofocal group; ODT: open door test; PBS: phone book search; RCS: reading computer screen; SD: standard deviation; ST: screwdriver test; SubRe: subtitles reading; SupRe: supermarket receipt.

**Table 5:** Comparisons among the light intensity levels for each light temperature (vertically) and among the light temperature levels for each light intensity (horizontally).

ADLs	Group	Lighting Settings	3000 K	4000 K	6000 K	*p*-Value (^a^ Repeated ANOVA/ ^b^ Friedman Test)
			Mean (95%CI) ^a^ Median (Interquartile Range) ^b^			
PBS	G1	25 fc	52.82 [39.59, 66.06]	50.48 [38.47, 62.50]	50.54 [39.81, 61.47]	0.924 ^a^
		50 fc	48.41 [37.69, 59.13]	54.11 [43.52, 64.69]	49.73 [41.41, 58.06]	0.127 ^a^
		75 fc	46.57 [36.48, 56.66]	56.70 [46.11, 67.28]	51.62 [40.12, 62.35]	**0.035** ^a,^*
	*p*-value (Repeated measures ANOVA)		0.192	0.250	0.802	
	G2	25 fc	29.50 [9.99, 49.00]	30.59 [12.46, 48.73]	33.23 [14.32, 52.15]	0.987 ^a^
		50 fc	29.01 [11.03, 46.98]	31.93 [13.60, 50.27]	38.29 [15.83, 60.75]	0.486 ^a^
		75 fc	40.22 [19.52, 60.92]	36.38 [16.53, 56.23]	49.26 [19.99, 78.53]	0.315 ^a^
	*p*-value (Repeated measures ANOVA)		0.079	0.514	**0.037** *	
	G3	25 fc	5.60 [−3.16, 14.35]	5.88 [−4.99, 16.75]	10.99 [0.71, 21.27]	0.365 ^a^
		50 fc	8.97 [−1.32, 19.25]	10.77 [−2.14, 23.67]	12.95 [1.29, 24.61]	0.630 ^a^
		75 fc	14.02 [1.63, 26.42]	21.74 [4.20, 39.28]	21.90 [5.64, 38.16]	0.073 ^a^
	*p*-value (Repeated measures ANOVA)		**0.023** *	**0.010** *	**0.016** *	
SupRe	G1	25 fc	56.63 [45.53, 67,72]	61.78 [52.18, 71.37]	59.70 [51.24, 68.17]	0.387 ^a^
		50 fc	58.51 [47.70, 69.33]	59.86 [51.65, 68.08]	66.39 [56.08, 76.70]	**0.046** ^a,^*
		75 fc	55.53 [46.12, 64.93]	63.41 [52.91, 73.90]	63.41 [52.91, 73.90]	**0.002** ^a,^*
	*p*-value (Repeated measures ANOVA)		0.565	0.534	0.156	
	G2	25 fc	53.19 [37.33, 67.05]	59.99 [41.35, 78.63]	56.43 [44.20, 68.67]	0.821 ^a^
		50 fc	66.93 [52.47, 81.39]	61.77 [50.93, 72.61]	64.05 [53.93, 74.18]	0.578 ^a^
		75 fc	63.62 [48.33, 78.90]	66.73 [51.93, 81.51]	70.47 [57.93, 83.01]	0.479 ^a^
	*p*-value (Repeated measures ANOVA)		**0.026** *	0.369	**0.011** *	
	G3	25 fc	32.91 [19.49, 46.33]	34.56 [16.99, 52.12]	36.94 [20.49, 53.40]	0.796 ^a^
		50 fc	38.35 [24.91, 51.79]	39.98 [25.67, 52.48]	43.33 [30.11, 56.55]	0.517 ^a^
		75 fc	41.80 [26.86, 55.73]	47.58 [35.91, 59.26]	46.58 [34.02, 59.13]	0.307 ^a^
	*p*-value (Repeated measures ANOVA)		0.203	**0.010** *	**0.033** *	
BR	G1	25 fc	90.89 [79.57, 102.21]	91.46 [80.38, 102.53]	92.34 [82.12, 102.57]	0.320 ^a^
		50 fc	94.15 [82.79, 105.51]	94.56 [84.45, 104.67]	93.52 [84.49, 103.55]	0.267 ^a^
		75 fc	95.52 [83.21, 107.82]	96.17 [85.88, 106.47]	94.09 [83.72, 104.45]	0.404 ^a^
	*p*-value (Repeated measures ANOVA)		0.093	**0.013** *	0.433	
	G2	25 fc	107.09 [91.99, 122.19]	99.74 [76.58, 122.89]	117.57 [103.15, 131.98]	0.173 ^a^
		50 fc	112.71 [97.67, 127.75]	108.48 [89.72, 127.24]	118.52 [104.00, 133.03]	0.679 ^a^
		75 fc	114.47 [98.76, 130.18]	112.62 [93.26, 131.98]	120.68 [109.39, 131.98]	0.546 ^a^
	*p*-value (Repeated measures ANOVA)		**0.009** *	0.082	0.651	
	G3	25 fc	72.49 [52.98, 92.01]	85.65 [64.45, 106.85]	90.79 [74.00, 107.58]	**0.027** ^a,^*
		50 fc	87.44 [73.50, 101.38]	90.80 [74.41, 107.18]	102.02 [89.67, 114.36]	**0.002** ^a,^*
		75 fc	99.15 [86.49, 11.82]	95.70 [79.00, 112.40]	104.29 [92.79, 115. 79]	0.108 ^a^
	*p*-value (Repeated measures ANOVA)		**<0.001** *	0.150	**0.005** *	
DR	G1	25 fc	67.19 [43.90, 77.41]	68.80 [51.33, 80.89]	59.68 [46.61, 68.17]	0.274 ^b^
		50 fc	68.56 [53.06, 86.23]	68.90 [55.22, 82.32]	67.68 [55.62, 78.93]	0.953 ^b^
		75 fc	66.79 [55.16, 79.60]	68.56 [61.55, 79.45]	66.40 [53.16, 86.14]	0.715 ^b^
	*p*-value (Friedman)		0.073	0.923	**0.012** *	
	G2	25 fc	44.76 [31.88, 57.65]	50.10 [30.71, 69.49]	50.86 [33.55, 68.18]	0.760 ^b^
		50 fc	57.73 [46.01, 69.45]	58.95 [38.48, 79.42]	61.23 [49.42, 73.04]	0.485 ^b^
		75 fc	63.43 [50.45, 76.40]	56.85 [37.97, 75.75]	60.77 [47.16, 74.38]	0.667 ^b^
	*p*-value (Repeated measures ANOVA)		**0.001** *	0.202	0.053	
	G3	25 fc	5.31 [2.28, 33.70]	27.32 [12.13, 42.50]	25.96 [12.07, 39.85]	0.170 ^b^
		50 fc	20.19 [8.80, 40.47]	28.26 [15.40, 41.01]	33.81 [22.08, 45.55]	0.246 ^b^
		75 fc	28.16 [18.85, 47.39]	34.22 [18.95 49.48]	36.54 [21.24, 51.85]	0.549 ^b^
	*p*-value (^a^ Repeated measures ANOVA/ ^b^ Friedman)		0.181 ^b^	0.358 ^a^	0.083 ^a^	
CM	G1	25 fc	74.21 [67.16, 81.26]	75.19 [68.20, 82.19]	70.40 [62.02, 78.79]	0.305 ^a^
		50 fc	77.31 [69.85, 84.76]	75.74 [69.22, 82.25]	72.46 [63.62, 81.29]	0.361 ^a^
		75 fc	72.24 [63.58, 80.90]	77.07 [70.54, 83.59]	74.33 [63.99, 84.66]	0.334 ^a^
	*p*-value (Repeated measures ANOVA)		0.184	0.493	0.352	
	G2	25 fc	78.83 [68.63, 89.02]	75.32 [61.90, 88.75]	77.81 [65.13, 90.48]	0.571 ^a^
		50 fc	73.98 [63.33, 84.62]	74.74 [66.14, 83.35]	76.12 [63.73, 88.52]	0.823 ^a^
		75 fc	73.91 [63.24, 84.58]	76.17 [64.27, 88.08]	76.67 [61.03, 92.31]	0.760 ^a^
	*p*-value (Repeated measures ANOVA)		0.264	0.898	0.783	
	G3	25 fc	54.31 [43.68, 64.95]	63.25 [50.59, 75.91]	66.01 [57.35, 74.67]	0.077 ^a^
		50 fc	63.42 [50.62, 76.22]	67.03 [55.59, 78.46]	67.26 [58.85, 75.67]	0.932 ^a^
		75 fc	61.25 [48.26, 74.24]	62.92 [49.03, 76.81]	66.84 [54.18, 79.51]	0.719 ^a^
	*p*-value (Repeated measures ANOVA)		0.095	0.280	0.860	
CES	G1	25 fc	70.13 [50.12, 79.33]	69.23 [54.82, 81.49]	68.34 [56.44, 80.35]	0.340 ^b^
		50 fc	74.34 [54.80, 81.67]	68.05 [51.20, 74.31]	68.48 [52.80, 75.87]	0.867 ^b^
		75 fc	72.90 [54.71, 78.67]	66.90 [49.66, 76.96]	69.24 [53.10, 81.28]	0.544 ^b^
	*p*-value (Friedman)		0.696	0.715	0.948	
	G2	25 fc	58.80 [48.03, 69.58]	58.78 [50.67, 74.34]	62.29 [48.65, 75.92]	0.693 ^b^
		50 fc	60.37 [51.42, 69.33]	66.54 [63.81, 73.41]	58.39 [45.59, 71.19]	0.485 ^b^
		75 fc	59.28 [51.32, 67.24]	66.31 [63.10, 69.59]	58.60 [44.01, 73.19]	0.693 ^b^
	*p*-value (^a^ Repeated measures ANOVA/ ^b^ Friedman)		0.800 ^a^	0.520 ^b^	0.404 ^a^	
	G3	25 fc	35.35 [21.09, 49.61]	43.82 [29.30, 58.34]	51.36 [38.30, 64.41]	0.149 ^b^
		50 fc	37.58 [22.85, 52.31]	48.83 [33.57, 64.09]	50.85 [39.83, 61.88]	0.059 ^b^
		75 fc	37.08 [22.20, 51.97]	43.94 [27.88, 60.00]	50.84 [38.16, 63.52]	0.149 ^b^
	*p*-value (Repeated measures ANOVA)		0.721	0.325	0.977	
RCS	G1	25 fc	89.63 [77.97, 101.29]	88.46 [75.91, 101.01]	87.32 [75.10, 99.55]	0.778 ^a^
		50 fc	87.47 [75.47, 99.47]	90.43 [78.13, 102.72]	86.89 [75.09, 98.68]	0.124 ^a^
		75 fc	88.91 [75.43, 102.39]	88.86 [75.21, 102.51]	86.46 [74.01, 98.92]	0.777 ^a^
	*p*-value (Repeated measures ANOVA)		0.721	0.763	0.803	
	G2	25 fc	112.01 [97.36, 126.65]	107.28 [77.49, 137.06]	114.10 [99.40, 128.81]	0.562 ^a^
		50 fc	110.24 [95.83, 124.65]	116.58 [81.16, 152.00]	114.66 [99.89, 129.42]	0.362 ^a^
		75 fc	113.73 [97.94, 129.52]	116.72 [102.14, 131.30]	118.91 [104.32, 33.51]	0.334 ^a^
	*p*-value (Repeated measures ANOVA)		0.411	0.575	0.276	
	G3	25 fc	104.04 [89.84, 118.25]	94.90 [68.62, 121.18]	98.77 [81.95, 115.59]	0.190 ^a^
		50 fc	106.37 [91.51, 121.24]	101.63 [83.38, 119.89]	102.78 [85.88, 119.68]	0.293 ^a^
		75 fc	103.82 [85.52, 122.12]	100.70 [82.99, 118.40]	110.00 [90.76, 129.23]	0.262 ^a^
	*p*-value (Repeated measures ANOVA)		0.630	0.366	**0.014** *	
SubRe	G1	25 fc	95.00 [85.63, 99.38]	97.50 [85.00, 100.00]	97.50 [81.25, 100.00]	0.174 ^b^
		50 fc	95.00 [88.75, 97.50]	97.50 [88.75, 100.00]	97.50 [88.75, 100.00]	**0.020** ^b,^*
		75 fc	95.00 [92.50, 97.50]	97.50 [95.00, 100.00]	97.50 [95.00, 100.00]	**0.004** ^b,^*
	*p*-value (Friedman test)		0.378	0.657	Β	
	G2	25 fc	93.75 [81.25, 100.00]	95.00 [85.00, 100.00]	95.00 [80.63, 100.00]	0.893 ^b^
		50 fc	87.50 [80.21, 97.50]	95.63 [87.50, 100.00]	97.50 [89.06, 100.00]	0.119 ^b^
		75 fc	92.50 [81.46, 96.25]	96.25 [72.50, 100.00]	100.00 [80.63, 100.00]	0.089 ^b^
	*p*-value (Friedman test)		0.662	0.648	0.120	
	G3	25 fc	91.25 [86.25, 98.75]	95.00 [72.50, 97.50]	97.50 [78.13, 100.00]	0.303 ^b^
		50 fc	91.88 [82.50, 98.13]	97.50 [85.00, 100.00]	96.25 [73.13, 100.00]	0.464 ^b^
		75 fc	93.75 [86.25, 98.75]	95.00 [77.50, 100.00]	95.00 [86.25, 99.38]	0.745 ^b^
	*p*-value (Friedman test)		0.905	0.165	0.386	
ODT	G1	25 fc	70.43 [50.12, 90.75]	73.71 [51.99, 95.43]	77.41 [62.24, 92.59]	0.828 ^a^
		50 fc	67.32 [40.20, 94.43]	82.47 [63.36, 101.58]	87.23 [64.72, 109.74]	**0.003** ^a,^*
		75 fc	73.76 [49.57, 97.95]	78.86 [57.58, 100.15]	90.78 [76.95, 104.60]	0.058 ^a^
	*p*-value (Repeated measures ANOVA)		0.594	0.192	0.128	
	G2	25 fc	84.92 [66.72, 103.12]	88.75 [77.85, 99.65]	82.13 [70.60, 93.66]	0.419 ^a^
		50 fc	87.81 [65.08, 110.53]	89.63 [79.57, 99.68]	96.63 [70.91, 122.35]	0.265 ^a^
		75 fc	87.98 [63.49, 112.47]	88.88 [78.39, 99.36]	89.47 [65.80, 113.14]	0.810 ^a^
	*p*-value (Repeated measures ANOVA)		0.674	0.824	0.276	
	G3	25 fc	71.00 [56.77, 85.24]	83.50 [66.50, 100.45]	77.04 [59.06, 5.02]	0.403 ^a^
		50 fc	76.82 [61.09, 92.55]	87.50 [73.65, 01.35]	79.03 [69.59, 88.46]	0.727 ^a^
		75 fc	79.07 [65.44, 92.71]	88.00 [77.11, 98.89]	87.12 [74.90, 99.35]	0.280 ^a^
	*p*-value (Repeated measures ANOVA)		0.333	0.193	0.131	
ST	G1	25 fc	64.17 [55.94, 72.41]	68.78 [59.78, 77.77]	64.17 [55.94, 72.41]	0.270 ^a^
		50 fc	69.30 [63.39, 75.22]	63.40 [55.42, 71.39]	69.31 [63.39, 75.22]	0.132 ^a^
		75 fc	72.20 [63.92, 80.48]	67.82 [59.07, 76.57]	72.20 [63.92, 80.48]	0.162 ^a^
	*p*-value (Repeated measures ANOVA)		0.053	0.170	0.053	
	G2	25 fc	58.88 [50.27, 67.48]	60.14 [43.89, 76.39]	60.96 [50.28, 71.63]	0.770 ^a^
		50 fc	65.46 [56.06, 74.86]	66.64 [53.96, 79.32]	66.72 [58.35, 75.09]	0.805 ^a^
		75 fc	61.33 [49.91, 72.75]	70.98 [62.92, 79.03]	67.94 [58.44, 77.44]	0.770 ^a^
	*p*-value (Repeated measures ANOVA)		0.328	0.147	0.397	
	G3	25 fc	54.10 [39.96, 68.24]	63.79 [48.25, 79.35]	64.85 [52.77, 76.93]	0.144 ^a^
		50 fc	57.69 [42.23, 73.16]	59.60 [49.19, 70.02]	62.18 [52.88, 71.47]	0.399 ^a^
		75 fc	68.32 [58.42, 78.22]	68.05 [58.36, 77.74]	67.50 [54.84, 80.17]	0.848 ^a^
	*p*-value (Repeated measures ANOVA)		0.018 *	0.323	0.431	

* *p* < 0.05 (in bold); ^a^ Repeated ANOVA/^b^ Friedman Test. Abbreviations: ADLs: activities of daily living, BR: book reading; CES: cellular entry search; CM: cellular message; DR: drops bottle reading; G1: trifocal group, G2: bifocal group, G3: monofocal group; ODT: open door test; PBS: phone book search; RCS: reading computer screen; ST: screwdriver test; SubRe: subtitles reading; SupRe: supermarket receipt.

**Table 6:** Subjective preference for lighting conditions during reading (number of subjects).

Temperature	3000 K			4000 K			6000 K		
Intensity	25 fc	50 fc	75 fc	25 fc	50 fc	75 fc	25 fc	50 fc	75 fc
G1	0	0	0	0	1	6	0	1	17
G2	0	0	0	0	2	2	0	1	20
G3	0	0	0	0	0	7	0	0	18

Abbreviation: fc: foot-candles, G1: trifocal group, G2: bifocal group, G3: monofocal group, and K: Kelvins.

**Table 7:** Light combinations presenting the best ADL scores and comparison with the most preferable light combination (6000 K, 75 fc).

ADLs	G1	G2	G3	*p*-Value (^a^ One-Way ANOVA, ^b^ Kruskal–Wallis Test)
	1. Best Light Temperature/Light Intensity 2. Mean ± SD OR Median (Interquartile Range) 3. *p*-Value (Comparison with 6000 K/75 fc)			
RCS	4000 K/50 fc 90.43 ± 26.27 0.226	4000 K/75 fc 116.72 ± 20.38 0.139	6000 K/75 fc 106.86 ± 31.12 NA	**0.036** ^a,^*
BR	4000 K/75 fc 96.17 ± 22.00 0.236	6000 K/50 fc 118.52 ± 21.60 0.466	6000 K/50 fc 102.19 ± 18.91 0.332	**0.027** ^a,^*
SubRe	6000 K/75 fc 97.50 [95.00, 100.00] NA	6000 K/75 fc 100.00 [83.75, 100.00] NA	6000 K/25 fc 97.50 [78.13, 100.00] 0.079	0.067 ^b^
ODT	6000 K/75 fc 90.78 ± 19.33 NA	6000 K/50 fc 96.63 ± 30.77 0.404	6000 K/75 fc 87.12 ± 17.09 NA	**0.044** ^a,^*
CM	4000 K/75 fc 77.07 ± 13.94 0.456	3000 K/25 fc 78.83 ± 16.05 0.449	6000 K/50 fc 67.26 ± 13.24 0.910	0.106 ^a^
ST	6000 K/75 fc 72.20 ± 18.19 NA	4000 K/75 fc 70.98 ± 10.48 0.587	3000 K/75 fc 68.32 ± 15.58 0.990	0.803 ^a^
CES	3000 K/75 fc 65.70 ± 22.78 0.677	6000 K/25 fc 64.22 ± 20.49 0.300	6000 K/25 fc 51.36 ± 20.55 0.838	0.181 ^a^
SupRe	6000 K/50 fc 66.09 ± 22.15 0.324	6000 K/75 fc 71.34 ± 18.05 NA	4000 K/75 fc 47.58 ± 18.38 0.809	**0.014** ^a,^*
DR	4000 K/75 fc 68.56 [61.55, 79.45] 0.857	4000 K/75 fc 68.18 [42.08, 74.15] 0.039 *	4000 K/75 fc 39.17 [17.97, 53.65] 0.901	**0.001** ^b,^*
PBS	4000 K/75 fc 56.70 ± 22.62 0.167	6000 K/75 fc 44.78 ± 41.56 NA	4000 K/75 fc 21.74 ± 29.03 0.738	**0.009** ^a,^*
eADLs	4000 K/75 fc 92.89 ± 17.92 0.475	6000 K/50 fc 108.48 ± 17.10 0.095	6000 K/50 fc 97.50 ± 18.47 0.648	0.079 ^a^
mADLs	6000 K/75 fc 62.06 ± 18.43 NA	4000 K/75 fc 71.35 ± 16.07 0.819	6000 K/75 fc 60.21 ± 17.26 NA	0.285 ^a^
dADLs	4000 K/75 fc 61.81 ± 19.93 0.324	6000 K/75 fc 58.82 ± 21.61 NA	4000 K/75 fc 35.74 ± 20.56 0.699	**0.003** ^a,^*
Total ADLs	4000 K/75 fc 74.98 ± 14.44 0.867	6000 K/75 fc 79.10 ± 15.59 NA	6000 K/75 fc 66.03 ± 16.61 NA	0.056 ^a^

* *p* < 0.05 (in bold); ^a^ One-Way ANOVA, ^b^ Kruskal–Wallis Test. Abbreviations: ADLs: activities of daily living, BR: book reading; CES: cellular entry search; CM: cellular message; DR: drops bottle reading; G1: trifocal group, G2: bifocal group, G3: monofocal group; NA: not applicable; ODT: open door test; PBS: phone book search; RCS: reading computer screen; SD: standard deviation; ST: screwdriver test; SubRe: subtitles reading; SupRe: supermarket receipt.

**Table 8:** Correlations between NEI-VFQ-39 and ADL scores taking all light combinations and groups into consideration.

	NEI-VFQ-39 Total Score		NEI-VFQ-39 Near Score	
	r	*p*-Value	r	*p*-Value
Total ADL score	0.219	<0.0001 *	0.063	0.2053
eADL score	−0.031	0.5362	−0.162	0.0012 *
mADL score	0.217	<0.0001 *	−0.009	0.8609
dADL score	0.234	<0.0001 *	0.112	0.0237 *
PBS	0.282	<0.0001 *	0.154	0.0023 *
SupRe	0.191	0.0001 *	0.061	0.2243
BR	0.018	0.715	−0.109	0.0306 *
DR	0.183	0.0002 *	0.084	0.0919
CM	0.196	0.0001 *	0.055	0.274
CES	0.201	0.0001 *	0.069	0.1716
RCS	−0.085	0.0942	−0.178	0.0004 *
SubRe	0.191	0.0002 *	0.070	0.1682
ODT	0.440	<0.0001 *	0.354	<0.0001 *
ST	0.222	<0.0001 *	0.125	0.0143 *

Spearman correlation, * *p* < 0.05 (in bold). Abbreviations: ADL: activity of daily living, BR: book reading; CES: cellular entry search; CM: cellular message; DR: drops bottle reading; ODT: open door test; PBS: phone book search; RCS: reading computer screen; ST: screwdriver test; SubRe: subtitles reading; SupRe: supermarket receipt.

## Data Availability

The authors intend to share deidentified participant data, including study information leaflets and written consent forms, for at least one year after the manuscript publication, acceptable in print form. The data are available upon request (email: panagiotopoulou.ek@gmail.com). All relevant data are in Greek.
